# Diagnostic accuracy of dynamic CZT-SPECT in coronary artery disease. A systematic review and meta-analysis

**DOI:** 10.1007/s12350-021-02721-8

**Published:** 2021-08-04

**Authors:** Mariska Panjer, Magdalena Dobrolinska, Nils R. L. Wagenaar, Riemer H. J. A. Slart

**Affiliations:** 1 Department of Nuclear Medicine and Molecular Imaging, Medical Imaging Center, University Medical Center Groningen, University of Groningen, Hanzeplein 1, 9700 RB Groningen, The Netherlands; 2 Department of Cardiology and Structural Heart Diseases, Medical University of Silesia in Katowice, Katowice, Poland; 3 Department of Biomedical Photonic Imaging, Faculty of Science and Technology, University of Twente, Enschede, The Netherlands; 4 Department of Nuclear Medicine, Ziekenhuis Groep Twente, Hengelo, The Netherlands

**Keywords:** CZT-SPECT, Dynamic flow, CAD, Accuracy, Systematic review, Meta-analysis

## Abstract

**Background:**

With the appearance of cadmium-zinc-telluride (CZT) cameras, dynamic myocardial perfusion imaging (MPI) has been introduced, but comparable data to other MPI modalities, such as quantitative coronary angiography (CAG) with fractional flow reserve (FFR) and positron emission tomography (PET), are lacking. This study aimed to evaluate the diagnostic accuracy of dynamic CZT single-photon emission tomography (SPECT) in coronary artery disease compared to quantitative CAG, FFR, and PET as reference.

**Materials and Methods:**

Different databases were screened for eligible citations performing dynamic CZT-SPECT against CAG, FFR, or PET. PubMed, OvidSP (Medline), Web of Science, the Cochrane Library, and EMBASE were searched on the 5th of July 2020. Studies had to meet the following pre-established inclusion criteria: randomized controlled trials, retrospective trails or observational studies relevant for the diagnosis of coronary artery disease, and performing CZT-SPECT and within half a year the methodological references. Studies which considered coronary stenosis between 50% and 70% as significant based only on CAG were excluded. Data extracted were sensitivity, specificity, likelihood ratios, and diagnostic odds ratios. Quality was assessed with QUADAS-2 and statistical analysis was performed using a bivariate model.

**Results:**

Based on our criteria, a total of 9 studies containing 421 patients were included. For the assessment of CZT-SPECT, the diagnostic value pooled analysis with a bivariate model was calculated and yielded a sensitivity of 0.79 (% CI 0.73 to 0.85) and a specificity of 0.85 (95% CI 0.74 to 0.92). Diagnostic odds ratio (DOR) was 17.82 (95% CI 8.80 to 36.08, *P* < 0.001). Positive likelihood ratio (PLR) and negative likelihood ratio (NLR) were 3.86 (95% CI 2.76 to 5.38, *P* < 0.001) and 0.21 (95% CI 0.13 to 0.33, *P* < 0.001), respectively.

**Conclusion:**

Based on the results of the current systematic review and meta-analysis, dynamic CZT-SPECT MPI demonstrated a good sensitivity and specificity to diagnose CAD as compared to the gold standards. However, due to the heterogeneity of the methodologies between the CZT-SPECT MPI studies and the relatively small number of included studies, it warrants further well-defined study protocols.

**Supplementary Information:**

The online version contains supplementary material available at 10.1007/s12350-021-02721-8.

## Introduction

Non-invasive imaging which assesses myocardial perfusion (MPI) plays an important role in the diagnosis of coronary artery disease (CAD).^1^ It provides information about myocardial function which is superior to anatomical assessment alone.^2^,^3^ MPI can be assessed by positron emission tomography (PET), single-photon emission computed tomography (SPECT), magnetic resonance imaging (MRI), or computed tomography (CT).

For years, invasive coronary angiography (CAG) has been considered a gold standard in the CAD diagnosis. However, a primary concern of this method is the fact that it provides only anatomical assessment. Over the last decade, invasively measured fractional flow reserve (FFR) became a reference for the assessment of hemodynamic significance of coronary lesion and it is a standard of care in patients with angiographically assessed stenosis > 50%.^1^,^3^ Notwithstanding the clinical value of CAG and FFR, the invasive character, risk of radiation, and injection of contrast are the main limitation in daily practice.

Within abovementioned noninvasive methods, PET is considered a gold standard. However, due to expensive tracer production and high costs, it is mainly limited to major academic centers. In contrast to PET, SPECT is less expensive, wider, and more easily available. Traditionally, SPECT MPI evaluates defects in myocardial perfusion through a semi-quantitative 5-point analysis. It provides only relative assessment of myocardial perfusion which is inferior to quantitative analysis. Furthermore, its diagnostic accuracy is also limited by the low resolution and artifacts.^4^ However, the recently introduced CZT camera overcomes these technical limitations. Due to higher resolution and shorter acquisition time, it not only improves the overall SPECT performance but also decreases radiation dose.^5^-^7^ Importantly, CZT cameras enable the quantification of myocardial blood flow (MBF) and myocardial flow reserve (MFR) which provides more accurate assessment of multivessel, left main, and microvascular disease than visual assessment or semi-quantitative perfusion evaluation.^8^ Therefore, we decided to focus on dynamic scans acquired with CZT-SPECT, based on which the quantitative, and thereby more precise, analysis of blood flow is available.

Recent trends in CZT-SPECT clinical application have led to the proliferation of studies investigating to estimate its diagnostic accuracy. However, the status of the comparison between dynamic CZT-SPECT and clinical diagnostic standards in functional assessment of CAD diagnosis is unclear so far. Until now, CZT-SPECT was mostly compared to CAG, which can only assess the anatomical significance of the stenosis. Therefore, the primary aim of this systematic review and meta-analysis was to evaluate current evidence on the diagnostic accuracy of dynamic CZT-SPECT compared to gold standards of functional CAD assessment.

## Materials and Methods

### Data Source and Study Selection

This systematic review and meta-analysis was performed according to PRISMA-DTA statement.^1^ Approval of Bioethical Committee was not necessary since it was obtained before publication of each included study. In order to identify suitable studies, the following databases were searched: PubMed, OvidSP (Medline), Web of Science, the Cochrane Library, and EMBASE. The MESH search strategy included the following key words: coronary artery disease or CAD or myocardial blood flow or myocardial ischemia and dynamic SPECT or cadmium zinc and telluride or CZT or D-SPECT and coronary angiography or CAG or fractional flow reserve or FFR or positron emission tomography or PET or coronary computed tomography angiography or CCTA or MRI or magnetic resonance imaging. Studies published up to the 5th of July 2020 were included in our analyses. We included all studies which reported to perform dynamic CZT-SPECT and within half a year the methodological references coronary angiography with or without FFR, positron emission tomography, magnetic resonance, or coronary computed tomography angiography. Studies which considered coronary stenosis between 50% and 70% as significant based only on CAG without FFR were excluded from this systematic review. The studies also required sufficient data to extract true positives, true negatives, false positives, and false negatives. The exclusion criteria were as follows: papers written in non-English, non-human trials, small patient numbers (*n* < 10), no full-text available, reviews, meta-analysis, editorials and case studies, inclusion of data duplicated in other studies. Screening was performed individually and independently by two reviewers; then, a consensus procedure followed any remaining inconsistencies were resolved by a third reviewer.

### Data Extraction and Quality Assessment

Data extracted contained first author, publication year, study type, patient number, index test (dynamic CZT-SPECT), acquisition protocol, comparator, type of radiotracer, true positives, true negatives, false positives, false negatives, type of coronary artery disease and patient characteristics such as age, sex, diabetes mellitus, body mass index, obesity, hypertension, hyperlipidaemia, and percentage of prior CAD.

To assess study quality, Quality Assessment of Diagnostic Accuracy Studies (QUADAS-2) was used. Currently, QUADAS-2 is recommended to evaluate the quality of diagnostic studies.^9^ The criteria examined by QUADAS-2 are risk of bias (selection bias, index test bias, reference test bias, and flow timing bias) and applicability concerns according to each of bias risk. Selection bias risk was deemed high if patient exclusion was not explained and intermediate/unknown if the patient population consisted of patients with known CAD. The index test in this study is considered to be dynamic CZT-SPECT. Index test bias was deemed low if image assessment was blinded from other diagnostic tests, final diagnosis and if a threshold was pre-specified. If a study did not meet one of the criteria risks, risk of bias was deemed intermediate/unknown. All studies fulfilling only one criterion were deemed high risk. When reference standard analysis was blinded from dynamic CZT-SPECT outcomes, the final diagnosis bias risk was considered low. Flow timing bias was more difficult to judge since there is no established cut-off for the right timing between both tests. Therefore, no study is considered to have high-risk flow timing bias because only studies performing dynamic CZT-SPECT and the comparator within 6 months were included. The quality assessment was performed independently by two reviewers (Panjer and Dobrolinska), disagreement was solved by discussion or a third reviewer (Wagenaar) and is summarized in applicability concerns. The applicability concerns were considered low if studies met our inclusion criteria concerning the index test and reference standard. However, applicability concerns were deemed intermediate/unknown if studies had a patient population with 100% known CAD.

### Statistical Analysis

Continuous variables were calculated as medians, and categorical variables were calculated as percentage. To assess the diagnostic accuracy of dynamic CZT-SPECT, sensitivity, specificity, positive likelihood ratio (PLR), negative likelihood ratio (NLR), and diagnostic odds ratio (DOR) were used. A bivariate model was used with 95% confidence intervals (95% CI). The bivariate model preserves the two-dimensional nature of the data throughout the analysis and takes into account the heterogeneity beyond chance between studies. In addition, a summary receiver-operating characteristic (SROC) curves were conducted to describe total diagnostic performance for both dynamic CZT-SPECT versus CAG, FFR and versus PET. The sROC curve is a plot of the true positive rate (sensitivity) as a function of the false positive rate (1-specificity). The heterogeneity was calculated as *I*^2^. An *I*^2^ higher than 50% was considered indicative of significant study heterogeneity. A *P* value <0.05 was considered statistically significant. A meta-analysis was performed using Review Manager (RevMan, Version 5.3. Copenhagen: The Nordic Cochrane Centre, The Cochrane Collaboration, 2014), and heterogeneity was calculated with Open Meta-Analyst (BROWN School of Public Health, Providence, RI, USA).

## Results

### Search Results and Study Characteristics

**Figure 1:**
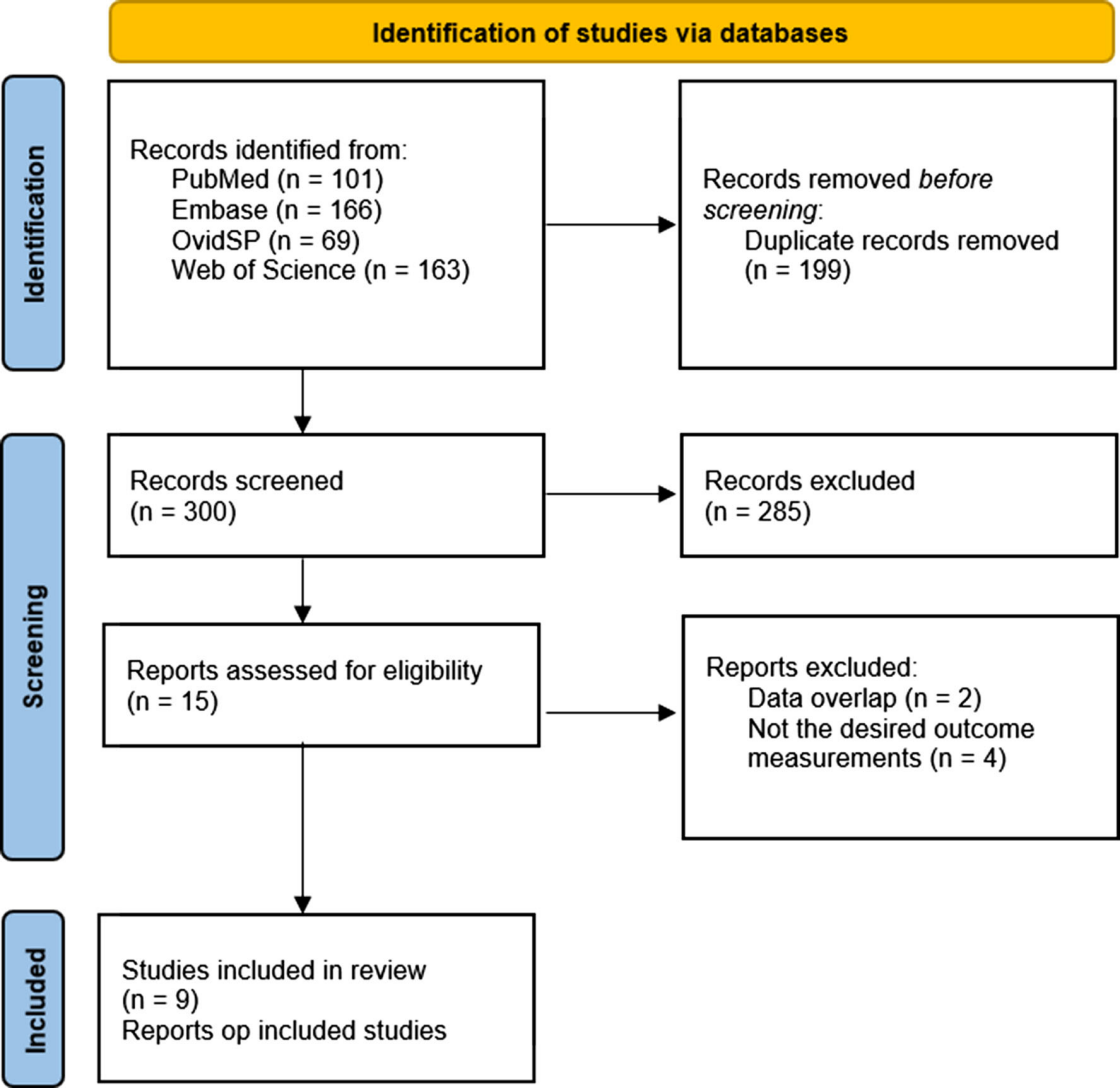
Flowchart of study selection process. Description of reviewing process for systematic review and meta-analysis

The results of study selection are visible in Fig. 1. After initial search, we identified 499 potentially relevant citations from which 15 studies were eligible for the primary study aim. No studies comparing CZT-SPECT to CCTA or MRI were eligible for inclusion. From these full-text 15 reviewed articles, nine that fulfilled the inclusion criteria were ultimately included for the analysis.

We included mostly single-center, prospective cohort studies (88.9%) which were published between 2015 and 2020. From nine studies, we encompassed altogether 421 patients. The sample size in the studies varied between 23 and 125 participants with median age of 66 years. Eight of the nine studies included patients with known CAD (89%), five studies included patients (55.5%) with previous myocardial infarction, and seven studies (77.8%) included more men than women. Moreover, every study included patients diagnosed with diabetes mellitus (Table 1).

**Table 1:** Study characteristics

Authors	Year	Country	Study design	Patients no.	Median age (yrs)	Male	DM	Obesity	HT	HCT	BMI (kg/m3)	Prior CAD	Prior MI
Agostini et al.^10^	2018	France	P	30	65	70%	33%	27%	67%	60%	NA	100%	NA
Bouallègue et al.^15^	2020	France	P	23	66	83%	17%	NA	30%	52%	26	100%	30%
Han et al.^16^	2018	Korea	P	34	60	74%	27%	NA	68%	74%	25	15%	11.8%
Li et al.^17^	2020	China	P	34	62	79.4	17.6	NA	55.9	20.6	24.6	100	0
Miyagawa et al.^12^	2017	Japan	P	69	69	67%	54%	NA	80%	55%	24	54%	15.8%
Nkoulou et al.^18^	2016	Switzerland	P	28	64	86%	32%	NA	61%	64%	28	NA	NA
Shiraishi et al.^19^	2015	Japan	P	55	74	44%	40%	NA	62%	53%	24	35%**	34.5%
Shiraishi et al.^20^	2019	Japan	R	125	75	58%	76%	NA	78%	66%	23	11%**	11.2%
Zavadovsky et al.^11^	2019	Russia/Italy	P	23	61	48%	22%	NA	87%	74%	29	52%	NA

*BMI*, body mass index; *CAD*, coronary artery disease; *DM*, diabetes mellitus; *HCT*, hypercholesterolemia; *HT*, hypertension; *MI*, myocardial infarction; *NA*, not available; *P*, prospective cohort; *R*, retrospective cohort

Within included studies, seven used as a comparator FFR (77.8%) and two used MPI-PET (22.2%). The definition of stenosis based on FFR was slightly different. Three studies defined FFR ≤ 0.8 (33.3%) and four FFR < 0.8 (44.4%) as significant. According to PET studies, Agostini et al. performed ^15^O-water PET for MPR assessment and included an FFR defined abnormal if ≤ 0.8.^10^

For the measurement of MPI, acquisition was performed with different types of CZT-SPECT cameras, six studies (66.7%) used Discovery NM 530c (GE Healthcare, Chicago, IL, USA), one study (11.1%) used Discovery NM/CT 570c (Alcyone technology, GE Healthcare, Haifa, Israel), and two studies (22.2%) used D-SPECT (Spectrum Dynamics, Palo Alto, California). Dual isotope administration was used in one study (11.1%), two studies (22.2%) used Thallium-201 as radiotracer, and six studies (66.7%) used Technetium-99m (Table 2). The mean or median radiation dose was reported in seven studies (77.8%) and it was higher in Thallium-201 studies. Stress/rest protocol was used in half of the studies (50%) and for the stress phase, the majority of the studies used adenosine (77.8%). Importantly, different software were used for the MBF quantification and measurement of MFR, including in-house software, with Corridor 4DM (INVIA, Ann Arbor, MI, USA) applied more frequently than others (Table 2). The method of diagnosing CAD was also different in the studies. Two studies^10^,^11^ (22.2%) performed a per-vessel analysis and the other seven (77.8%) performed a per-patient analysis. One study (11.1%) diagnosed triple-vessel disease (TVD).^12^

**Table 2:** Technical aspects of dynamic CZT-SPECT studies included in meta-analysis

Authors	Index test	Mean total effective dose (mSv)	Rest dose (MBq)	Stress dose (MBq)	Stress injection protocol	CZT cut-off value
Agostini et al.^10^	Rest/stress Tc MPI	8.73	3MBq/kg	9MBq/kg	Regadenoson (400 μg)	MFR < 2.1
Bouallègue et al.^15^	Rest/stress Tc MPI	8.4	220-280	645-730	Dipyridamole 0.75 mg/kg	MFR < 2
Han et al.^16^	Stress Tl/rest Tc MPI	9.13	222–370	44–66	Adenosine (140 mcg/kg/min)	MFR ≤ 2.0
Li et al.^17^	Rest/stress MIBI	N.A.	555	925	Adenosine (140 mcg/kg/min)	MFR < 2.12
Miyagawa et al.^12^	Stress/rest Tc MPI	6.34	3MBq/kg	9 MBq/kg	Adenosine (160 mcg/kg/min)	MFR < 1.3
Nkoulou et al.^18^	Stress/rest Tc MPI	N.A.	~1000	330±33	Adenosine (140 mcg/kg/min)	MFR <1.26
Shiraishi et al.^19^	Stress/rest Tl MPI	15.5	50–60	50–60	Adenosine 0.120 mg/kg/min)	MFR ≤ 1.5
Shiraishi et al.^20^	Stress/rest Tl MPI	15.5	50–60	50–60	Adenosine 0.120 mg/kg/min)	MFR < 2.66
Zavadovsky et al.^11^	Rest/stress Tc MPI	8.02	3MBq/kg	9MBq/kg	Adenosine (140 mcg/kg/min)	MFR ≤ 1.48

Authors	CZT-SPECT	Comparator	Definition of comparator cut-off value	Time between tests	Analysis Software
Agostini et al.^10^	D-SPECT	^15^O-Water PET	MFR < 2	30 d	Corridor4DM (INVIA Medical Imaging Solutions)
Bouallègue et al.^15^	Discovery NM 530c	FFR	FFR ≤ 0.8	4 w	In-house software
Han et al.^16^	Discovery NM 530c	FFR	FFR ≤ 0.8	90 d	Corridor4DM (INVIA Medical Imaging Solutions)
Li et al.^17^	D-SPECT	FFR	FFR < 0.8	3 days	Corridor4DM (INVIA Medical Imaging Solutions)
Miyagawa et al.^12^	Discovery NM 530c	FFR	FFR < 0.8	2 m	NA
Nkoulou et al.^18^	Discovery NM 530c	^13^NH_3_-ammonia PET	MFR < 2.0	12 weeks	PMOD software package (version 3.1; PMOD Technologies Ltd.)
Shiraishi et al.^19^	Discovery NM 530c	FFR	FFR < 0.8	30 d	In-house software
Shiraishi et al.^20^	Discovery NM 530c	FFR	FFR < 0.8	90 d	AZE Virtual Place Hayabusa (AZE, Ltd., Tokyo, Japan)
Zavadovsky et al.^11^	Discovery NM 570c	FFR	FFR ≤ 0.8	7 d	Corridor4DM (INVIA Medical Imaging Solutions)

*CZT-SPECT*, cadmium-zinc-telluride single-photon emission tomography; *FFR*, fractional flow reserve; *MFR*, myocardial flow rate; *MPI*, myocardial perfusion imaging; *NA*, not available; *PET*, positron emission tomography; *Tc*, technetium-99m; *Tl*, Thallium-201

### Methodological Quality of Included Studies

**Figure 2:**
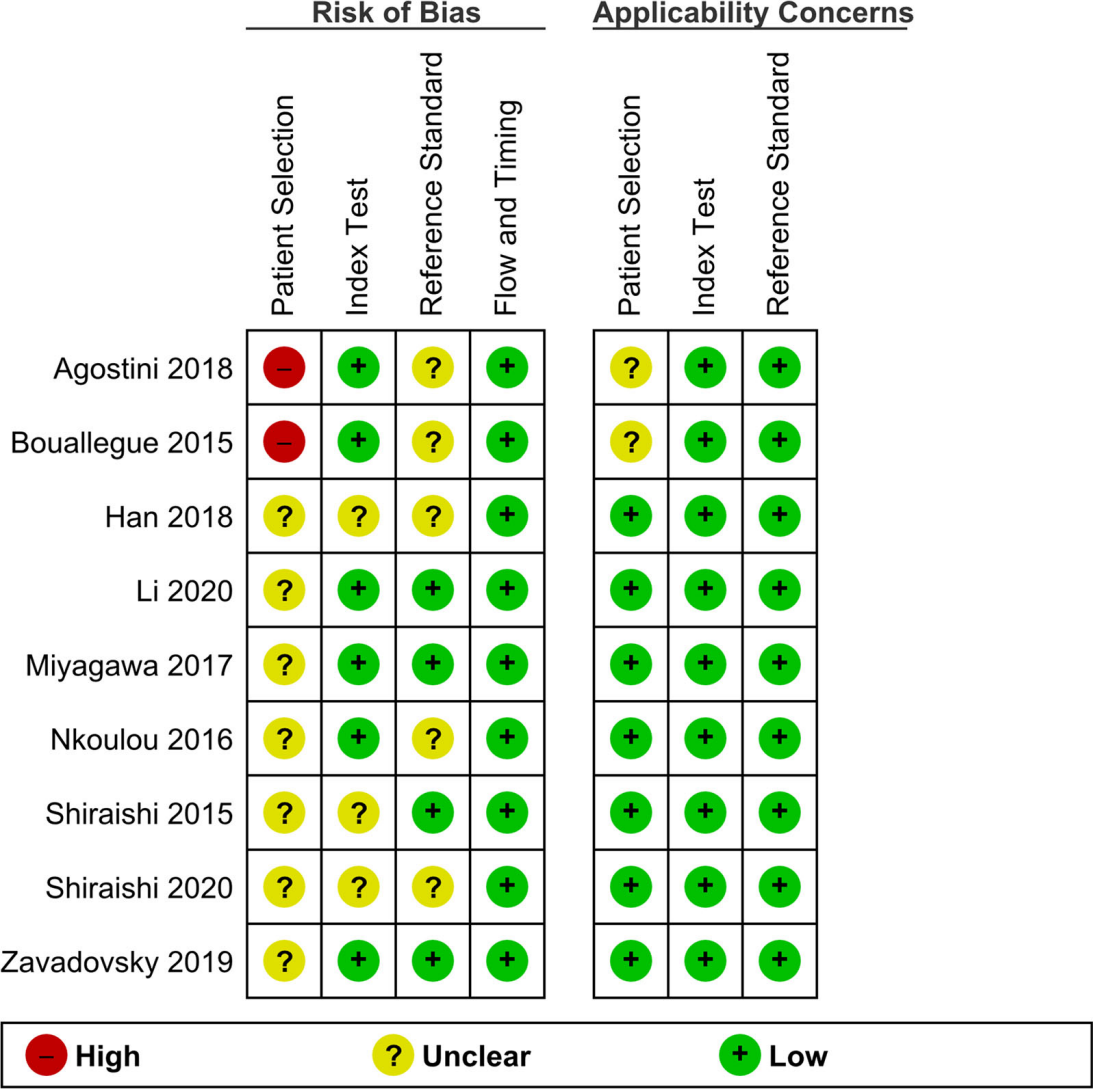
Quadas-2 risk of bias and applicability concerns summary. The summary of study inclusion criteria defined as low, unclear, and high, in terms of bias and applicability

Figure 2 summarizes the risk of bias and applicability concerns of the included studies. The applicability concerns were considered overall low. The domain which showed unclear risk of bias was ‘patients’ selection’ due to inclusion of patients with previously known CAD. Only two studies (22.2%) reported the reference standard to be assessed blinded, and all other studies did not specify blinding the data.

### Diagnostic Accuracy of Dynamic CZT-SPECT

**Figure 3:**
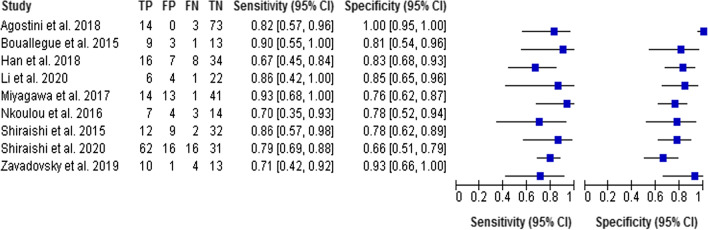
Forest plot for sensitivity and specificity. *TP*, true positive; *FP*, false positive; *FN*, false negative; *TN*, true negative; *CI*, confidence interval

**Figure 4:**
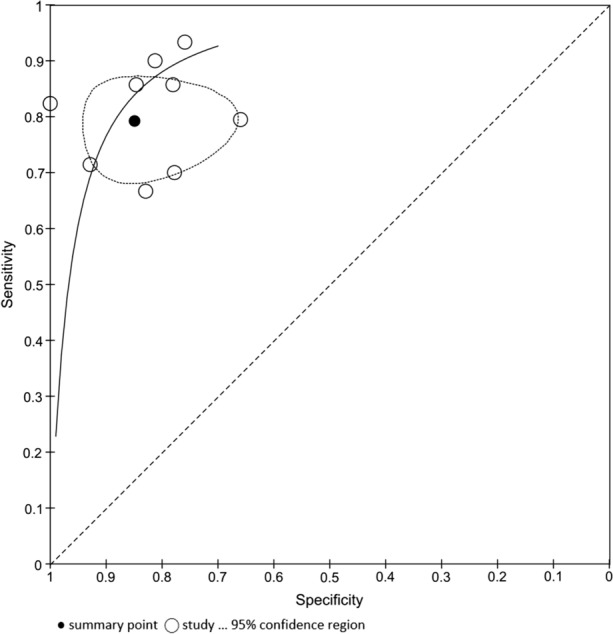
Summary Receiver Operating Curve (SROC) of included studies

**Figure 5:**
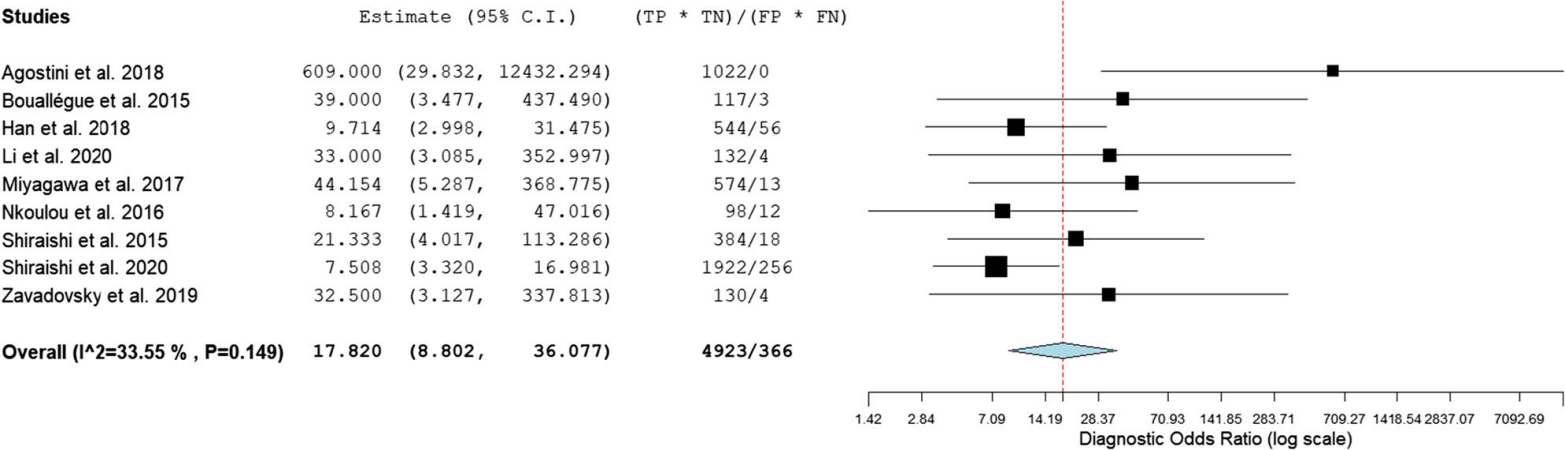
Forest plots for diagnostic odds ratio. *C.I.*, confidence interval; *TP*, true positive; *TN*, true negative; *FP*, false positive; *FN*, false negative

**Figure 6:**
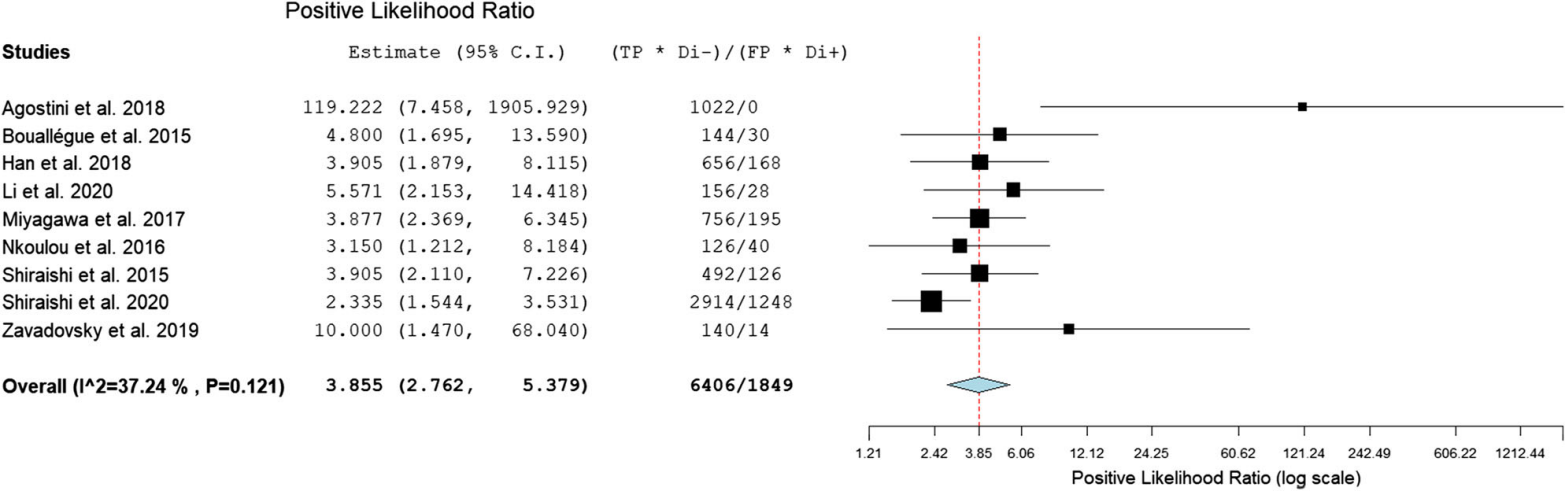
Forest plots for positive likelihood ratio. *C.I.*, confidence interval; *TP*, true positive; *FP*, false positive; *Di−*, disease absent; *Di+*, disease present

**Figure 7:**
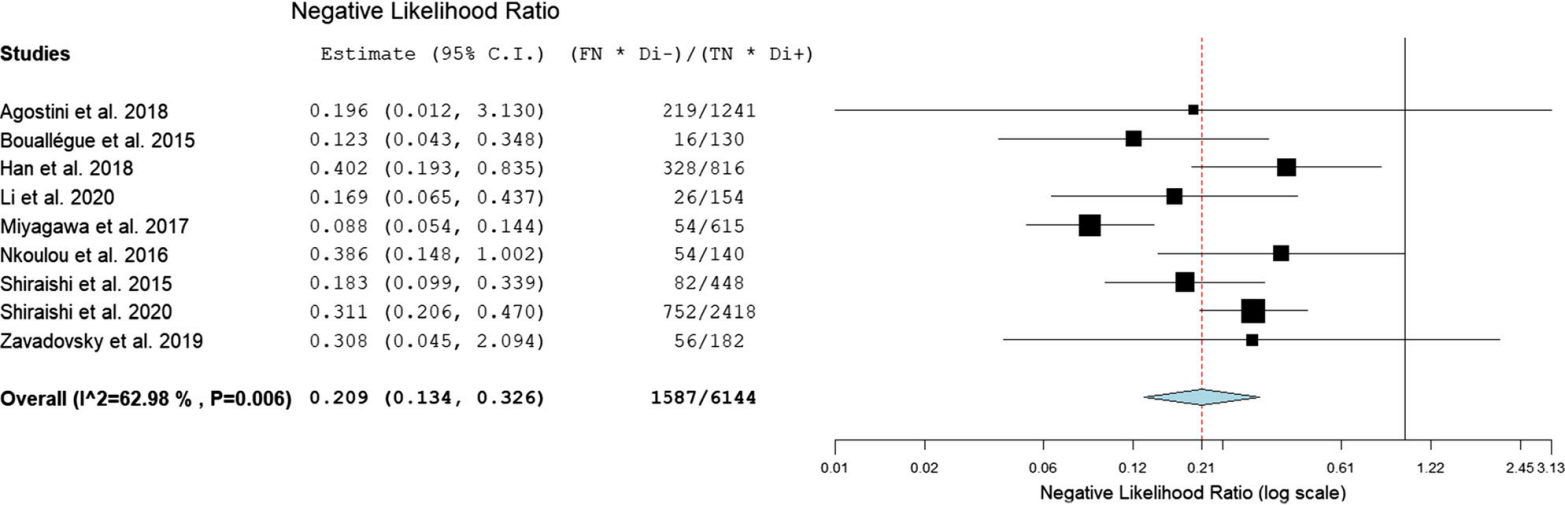
Forest plots for negative likelihood ratio. *C.I.*, confidence interval; *FN*, false negative; *TN*, true negative; *Di−*, disease absent; *Di+*, disease present

For the assessment of CZT-SPECT against both comparators PET and FFR, the diagnostic value pooled analysis with a bivariate model was calculated and yielded a sensitivity of 0.79 (95% CI 0.73 to 0.85) and a specificity of 0.85 (95% CI 0.74 to 0.92) (Fig. 3, Table 3). An analysis of diagnostic accuracy is summarized on a SROC curve (Fig. 4). The subgroup that compared CZT-SPECT to FFR only (*n* = 7) demonstrated a similar sensitivity of 0.80 (95% Cl 0.73 to 0.85) but lower specificity of 0.78 (Cl 0.71 to 0.83) as compared to the whole group. Diagnostic odds ratio (DOR) was 17.82 (95% CI 8.80 to 36.08, *P* < 0.001). Positive likelihood ratio (PLR) and negative likelihood ratio (NLR) were 3.86 (95% CI 2.76 to 5.38, *P* < 0.001) and 0.21 (95% CI 0.13 to 0.33, *P* < 0.001), respectively. Results are summarized in Figs. 5, 6, and 7. DOR, PLR, and NLR were calculated by a diagnostic random effects model. A Spearman’s rank correlation test showed no evidence of a threshold effect, Spearman’s correlation coefficient = − 0.3264 (*P* = 0.3914).

**Table 3:** Features of diagnostic accuracy from dynamic CZT-SPECT studies included in meta-analysis

Authors	Sensitivity	Specificity	Positive likelihood ratio	Negative likelihood ratio	Diagnostic odds ratio	True positive	True negative	False positive	False negative	PPV	NPV
Agostini et al.^10^	0.83	1	–	0.18	–	14	73	0	3	100	96
Bouallègue et al.^15^	0.89	0.82	4.80	0.12	39	9	13	3	1	75	93
Han et al.^16^	0.67	0.83	3.90	0.40	9.7	16	34	7	8	70	81
Li et al.^17^	0.86	0.85	5.57	0.17	33	6	22	4	1	60	96
Miyagawa et al.^12^	0.93	0.76	3.99	0.09	44.2	14	41	13	1	52	98
Nkoulou et al.^18^	0.70	0.78	3.15	0.39	8.17	7	14	4	3	64	82
Shiraishi et al.^20^	0.79	0.66	2.33	0.31	7.5	62	31	16	16	79	66
Shiraishi et al.^19^	0.86	0.78	3.90	0.18	21.3	12	32	9	2	57	94
Zavadovsky et al.^11^	0.69	0.93	10.00	0.31	32.5	10	13	1	4	91	76

*PPV*, positive predictive value; *NPV*, negative predictive value

### Heterogeneity Analysis

Heterogeneity of this study was substantial for NLR, *I*^2^ is 63% (*P* < 0.006). For PLR and DOR, heterogeneity was moderate but not significant, 37% (*P* < 0.121) and 34% (*P* = 0.149), respectively.

## Discussion

The high-sensitivity dedicated cardiac CZT-SPECT camera allows dynamic acquisition of tomographic images suitable for in vivo assessment of radiotracer kinetics and opens up a new era for myocardial flow and flow reserve measurement using SPECT imaging.

Our analysis was focused on the diagnostic accuracy of dynamic CZT-MPI SPECT. Based on this literature search and analysis, dynamic CZT-SPECT demonstrated a good sensitivity and specificity to diagnose coronary artery disease as compared to gold standards of functional and (quantitative) anatomical assessment.

The slightly reduced sensitivity of 79% in the current study might be explained by substantial discordance between FFR and MFR techniques. Notwithstanding the excellent diagnostic value of FFR, it quantifies the pressure gradient across the stenosis, but does not reflect microcirculation. As opposed to FFR, MFR reflects flow in epicardial arteries and microvasculature.^15^ It should be stressed that abnormal MFR with insignificant FFR indicates microvascular dysfunction or diffuse CAD.^15^,^16^ Therefore, FFR and MFR are not equivalent.^15^ Importantly, when CZT-SPECT MPI was compared to gold standard ^15^O-water PET, it gained an excellent accuracy.^10^ According to current guidelines, a diameter stenosis > 50% should be verified with FFR. Moreover, anatomical assessment does not reflect the MFR calculated with CZT-SPECT MPI. Therefore, we decided to include only studies which defined the comparator according to current gold standards, which includes functional assessment. This may explain a higher diagnostic odds ratio and specificity in the current meta-analysis as compared to CZT-SPECT MPI study of Nudi et al.^13^ This previous systematic review and meta-analysis on CZT-SPECT MPI has reported satisfactory sensitivity (0.84) and decreased specificity (0.69) of CZT-SPECT MPI.^13^ However, this decreased specificity might be due to the fact that MPI was compared to the % diameter stenosis, which is only an anatomical measurement. Also, different comparator cut-off values of % diameter stenosis were used, including 50% stenosis, and the included studies were mainly exist of static and not dynamic CZT-SPECT MPI scans.^13^

MPI is an important diagnostic tool for patients with known or suspected CAD. Therefore, a major effort was made to improve conventional SPECT MPI and replace it by CZT-SPECT camera.

The CZT-SPECT enables quantitative perfusion assessment due to its ultrafast signal processing. With dynamic cardiac acquisition, it is possible to detect the first-pass blood perfusion of tracer and its extraction into the myocardium allowing quantification of myocardial blood flow using dedicated compartment models. The CZT detector functions as a semiconductor with direct conversion of gamma radiation to an electric signal. This mechanism results in better spatial resolution and higher sensitivity, resulting in shorter acquisition time and/or lower radiation exposure. This offers superior performance advantages over sodium iodine detectors in Anger cameras.^14^

As a novelty, which enables to quantitatively measure myocardial perfusion by SPECT as MBF and MFR, it gained further interest. Taking both indices into consideration, it was previously found that MFR yields higher diagnostic value as compared to MBF.^10^

### Study Limitations

The main limitation of this systematic review and meta-analysis, additional to the typical limitations for this kind of analysis, is a relatively small number of included studies and patients and heterogeneity within comparators.

Heterogeneity between studies was substantial in terms of negative risk ratio and might be explained by effect modifiers. Firstly, different comparators were used. Moreover, there was also a diversity within study protocols, CZT cameras, radiotracers, dose administration, and software packages used for MPI CZT SPECT calculation. Furthermore, in each study, a different cut-off value for dynamic SPECT-MPI was set.

The present meta-analysis has mainly examined the CZT-MPI versus FFR. However, for further validation, CZT MPI should be compared to equivalent methods, including gold standard ^15^O-water PET or invasive coronary flow reserve (CFR) and index of microvascular resistance (IMR).

## Conclusion

Overall, dynamic CZT-SPECT MPI has a good diagnostic sensitivity and specificity for CAD diagnosis as compared to gold standards used in clinical practice. However, due to the heterogeneity of the methodologies between the CZT-SPECT MPI studies and the gold standards, and the relatively small number of included studies, it warrants further well-defined research protocols.

## New Knowledge Gained

The fact that CZT-SPECT MPI shows good diagnostic sensitivity and specificity for diagnosis of CAD indicates its value in everyday clinical practice.

## Supplementary Information

Below is the link to the electronic supplementary material.

Supplementary file1 (DOCX 26 kb)

Supplementary file2 (PPTX 214 kb)
