# Supplementary material for: Diagnostic accuracy of dynamic CZT-SPECT in coronary artery disease. A systematic review and meta-analysis
Source: J Nucl Cardiol. 2021 Aug 4;29(4):1686–97. doi: 10.1007/s12350-021-02721-8 (PMC9345813; doi:10.1007/s12350-021-02721-8)
Supplement: Supplementary file 1 — Supplementary file1 (DOCX 26 kb) [file 12350_2021_2721_MOESM1_ESM.docx]

Supplementary material

S1. PRISMA statement

| **SECTION/TOPIC** | **REPORTED** |
| --- | --- |
| **Title** | |
| Title | + |
| **Abstract** | |
| Structured summary | + |
| **Introduction** | |
| Rationale | + |
| Objectives | + |
| **Methods** | |
| Eligibility criteria | + |
| Information sources | + |
| Search | + |
| Study selection | + |
| Data collection process | + |
| Data items | + |
| Risk of bias in individual studies | + |
| Effect measures | - |
| Synthesis method | + |
| Reporting bias assessment | + |
| Certainty assessment | + |
| **Results** |  |
| Study selection | **+** |
| Study characteristics | + |
| Risk of bias within studies | + |
| Results of individual studies | + |
| Synthesis of results | + |
| Risk of bias across studies | + |
| Certainty of evidence | + |
| **Discussion** |  |
| Summary of evidence | + |
| Limitations | + |
| Conclusions | + |
| **Funding** | NA |
| Funding | NA |

S2. Search strategy

The MESH search strategy: ("coronary artery disease"[MeSH Terms] OR coronary artery disease[Text Word] OR myocardial blood flow[Text Word] OR MBF[Text Word] OR "myocardial ischemia"[MeSH Terms] OR myocardial ischemia[Text Word]) AND (dynamic SPECT[Text Word] OR "CdZnTe" [Supplementary Concept] OR cadmium zinc and telluride[Text Word] OR CZT[Text Word] OR D-SPECT[Text Word]) AND ("Coronary Artery Disease"[Mesh] OR coronary angiography[Text Word] OR CAG[Text Word] OR "Fractional Flow Reserve, Myocardial"[Mesh] OR fractional flow reserve[Text Word] OR FFR[Text Word] OR "Positron-Emission Tomography"[Mesh] OR positron-emission tomography[Text Word] OR PET[Text Word] OR coronary computed tomography angiography[Text Word] OR CCTA[Text Word] OR "magnetic resonance imaging"[MeSH Terms] OR magnetic resonance imaging[Text Word] OR MRI[Text Word])

S3. Exclusion summary

| Acampa et al.[1] | Not the desired outcome measurements |
| --- | --- |
| Acampa et al.[2] | Not the desired outcome measurements |
| Gimelli et al.[3] | Did not meet our inclusion criteria |
| Guibbini et al.[4] | Not the desired outcome measurements |
| Hyafil et al.[5] | Not the desired outcome measurements |
| Souza et al.[6] | Did not meet our inclusion criteria |

References:

1. Acampa W, Assante R, Mannarino T, et al (2020) Low-dose dynamic myocardial perfusion imaging by CZT-SPECT in the identification of obstructive coronary artery disease. Eur J Nucl Med Mol Imaging. https://doi.org/10.1007/s00259-019-04644-6

2. Acampa W, Zampella E, Assante R, et al (2020) Quantification of myocardial perfusion reserve by CZT-SPECT: A head to head comparison with 82Rubidium PET imaging. J Nucl Cardiol. https://doi.org/10.1007/s12350-020-02129-w

3. Gimelli A, Liga R, Duce V, et al (2017) Accuracy of myocardial perfusion imaging in detecting multivessel coronary artery disease: A cardiac CZT study. J Nucl Cardiol. https://doi.org/10.1007/s12350-015-0360-8

4. Giubbini R, Bertoli M, Durmo R, et al (2019) Comparison between N13NH3-PET and 99mTc-Tetrofosmin-CZT SPECT in the evaluation of absolute myocardial blood flow and flow reserve. J Nucl Cardiol. https://doi.org/10.1007/s12350-019-01939-x

5. Hyafil F, Chequer R, Sorbets E, et al (2020) Head-to-head comparison of the diagnostic performances of Rubidium-PET and SPECT with CZT camera for the detection of myocardial ischemia in a population of women and overweight individuals. J Nucl Cardiol Off Publ Am Soc Nucl Cardiol 27:755–768. https://doi.org/10.1007/s12350-018-01557-z

6. de Souza AC do AH, Gonçalves BKD, Tedeschi AL, Lima RSL (2019) Quantification of myocardial flow reserve using a gamma camera with solid-state cadmium-zinc-telluride detectors: Relation to angiographic coronary artery disease. J Nucl Cardiol. https://doi.org/10.1007/s12350-019-01775-z
