# Supplementary material for: Diagnostic accuracy of dynamic CZT-SPECT in coronary artery disease. A systematic review and meta-analysis
Source: J Nucl Cardiol. 2021 Aug 4;29(4):1686–97. doi: 10.1007/s12350-021-02721-8 (PMC9345813; doi:10.1007/s12350-021-02721-8)
Supplement: Supplementary file 2 — Supplementary file2 (PPTX 214 kb) [file 12350_2021_2721_MOESM2_ESM.pptx]

## Slide 1
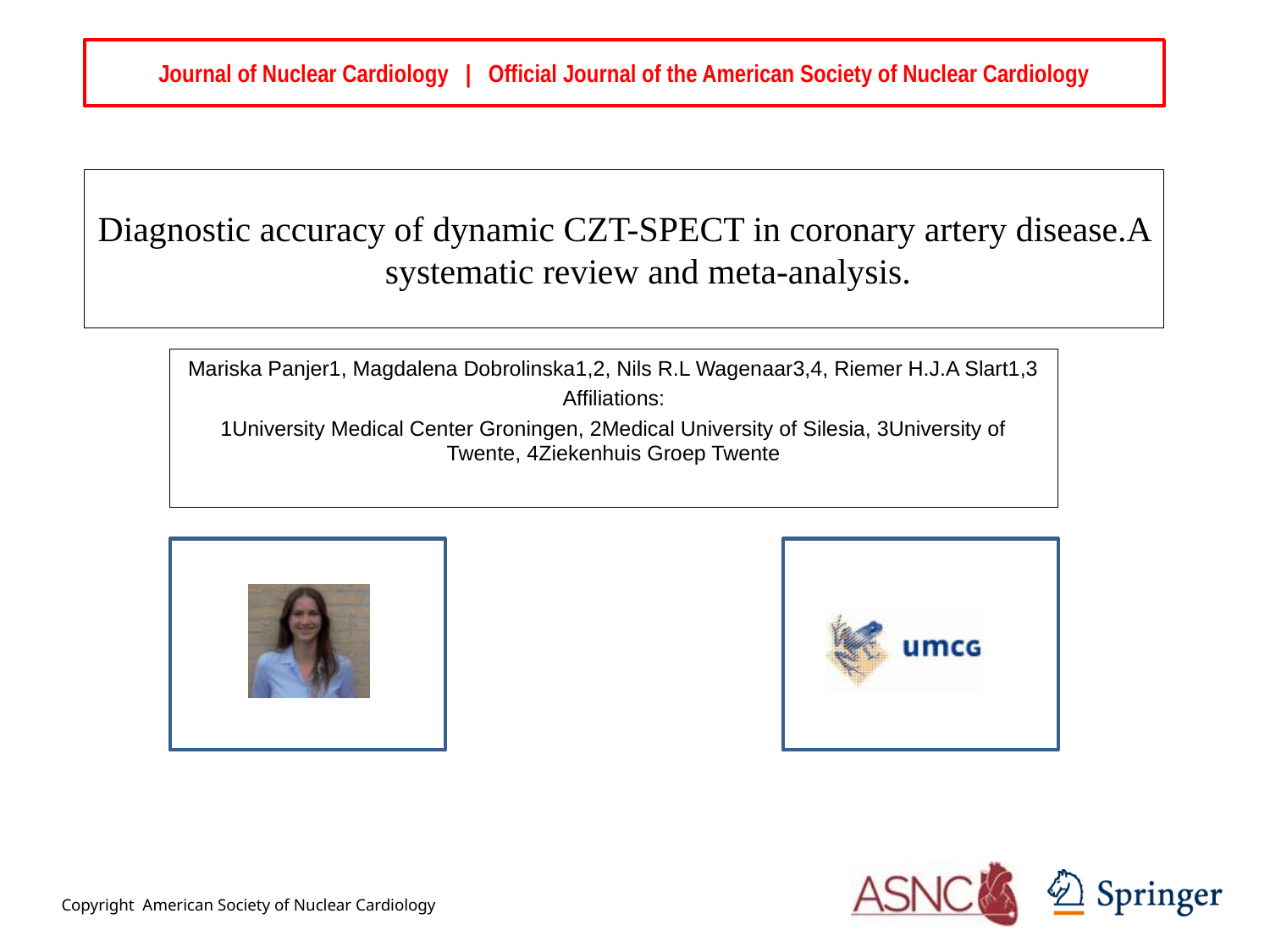

Journal of Nuclear Cardiology | Official Journal of the American Society of Nuclear Cardiology
# Diagnostic accuracy of dynamic CZT-SPECT in coronary artery disease.A systematic review and meta-analysis.
Mariska Panjer1, Magdalena Dobrolinska1,2, Nils R.L Wagenaar3,4, Riemer H.J.A Slart1,3
Affiliations:
1University Medical Center Groningen, 2Medical University of Silesia, 3University of Twente, 4Ziekenhuis Groep Twente
Copyright American Society of Nuclear Cardiology

## Slide 2
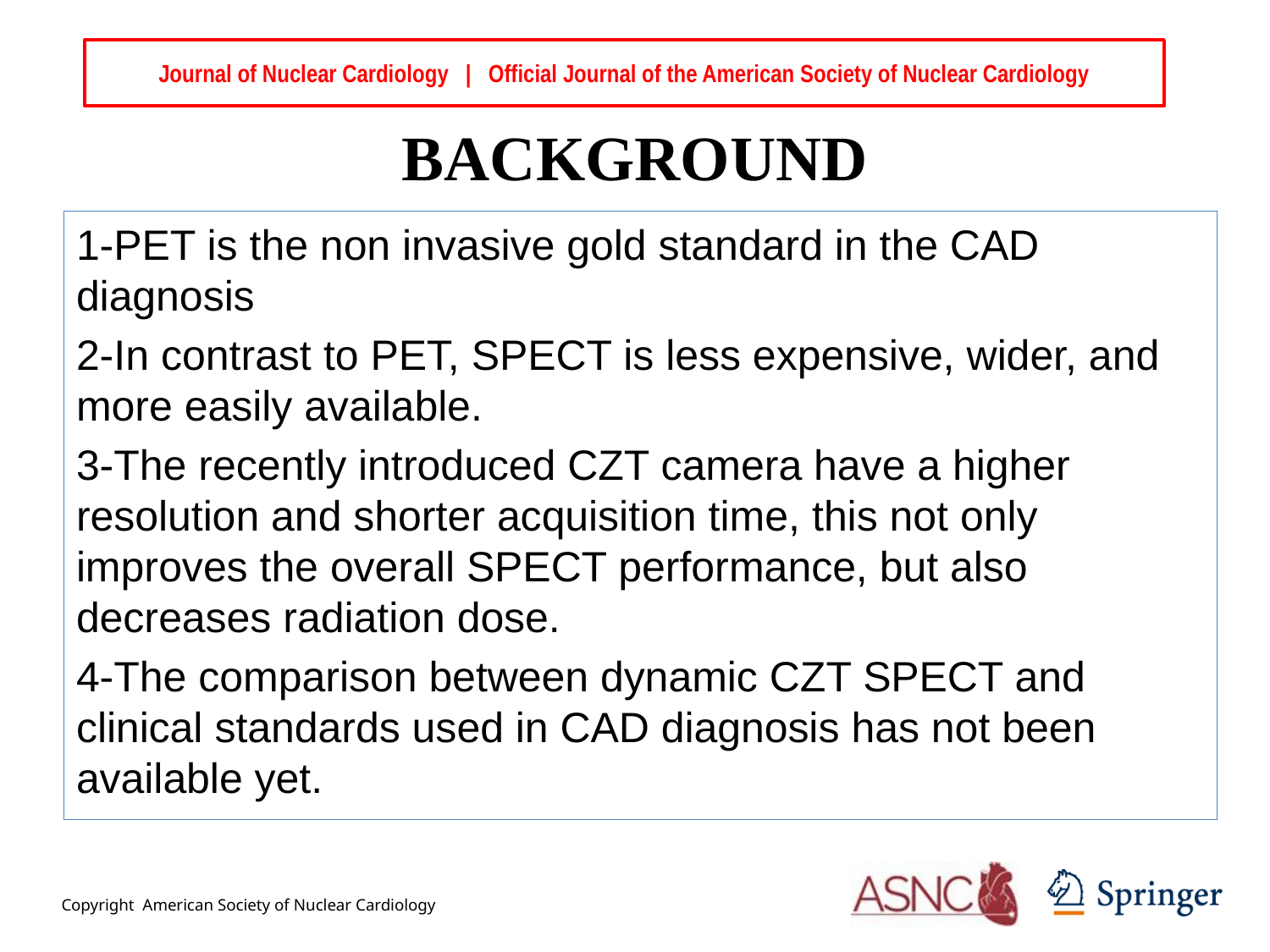

Journal of Nuclear Cardiology | Official Journal of the American Society of Nuclear Cardiology
# BACKGROUND
1-PET is the non invasive gold standard in the CAD diagnosis
2-In contrast to PET, SPECT is less expensive, wider, and more easily available.
3-The recently introduced CZT camera have a higher resolution and shorter acquisition time, this not only improves the overall SPECT performance, but also decreases radiation dose.
4-The comparison between dynamic CZT SPECT and clinical standards used in CAD diagnosis has not been available yet.
Copyright American Society of Nuclear Cardiology

## Slide 3
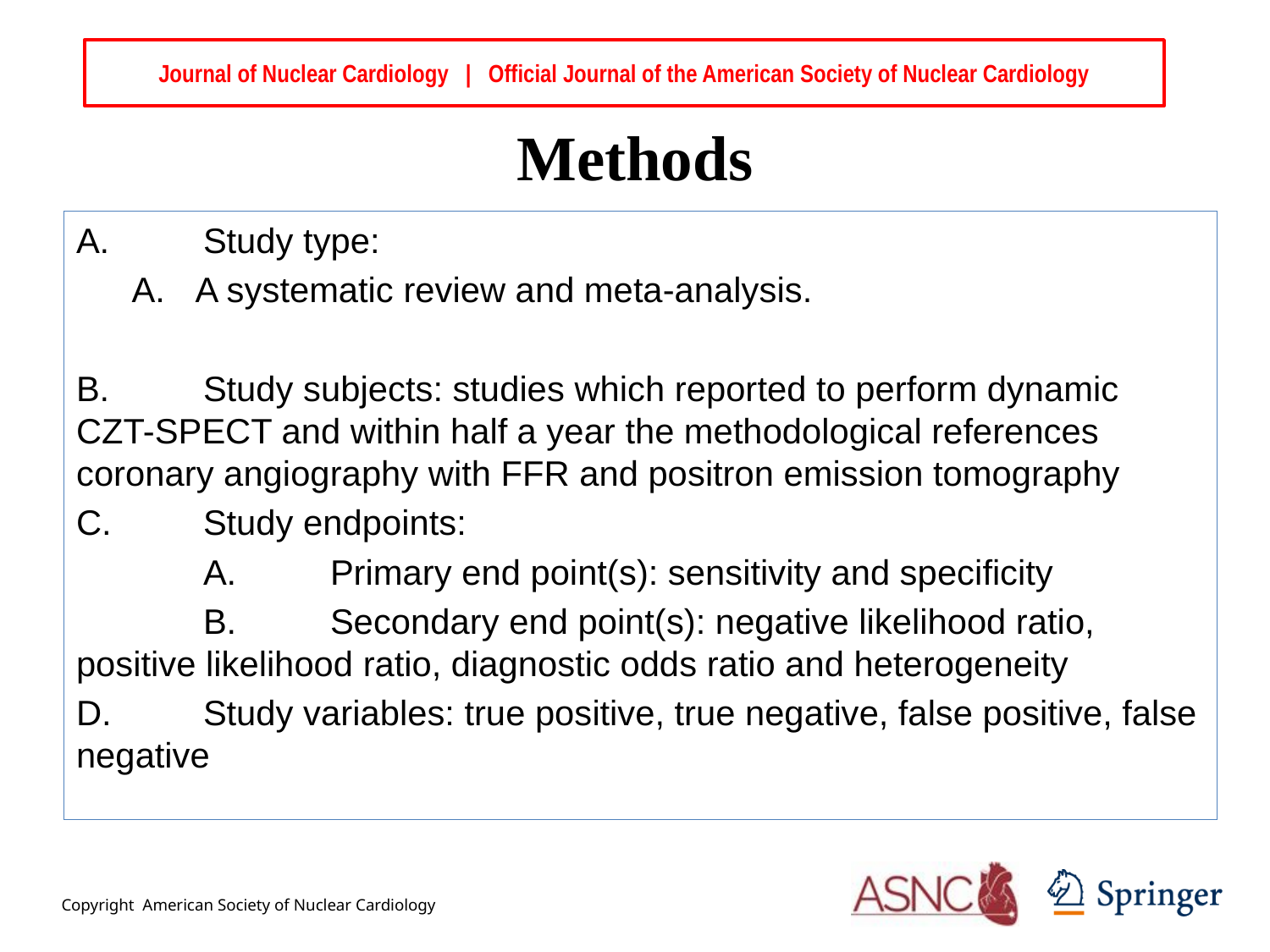

Journal of Nuclear Cardiology | Official Journal of the American Society of Nuclear Cardiology
# Methods
A.	Study type:
A systematic review and meta-analysis.
B.	Study subjects: studies which reported to perform dynamic CZT-SPECT and within half a year the methodological references coronary angiography with FFR and positron emission tomography
C.	Study endpoints:
	A.	Primary end point(s): sensitivity and specificity
	B.	Secondary end point(s): negative likelihood ratio, positive likelihood ratio, diagnostic odds ratio and heterogeneity
D.	Study variables: true positive, true negative, false positive, false negative
Copyright American Society of Nuclear Cardiology

## Slide 4
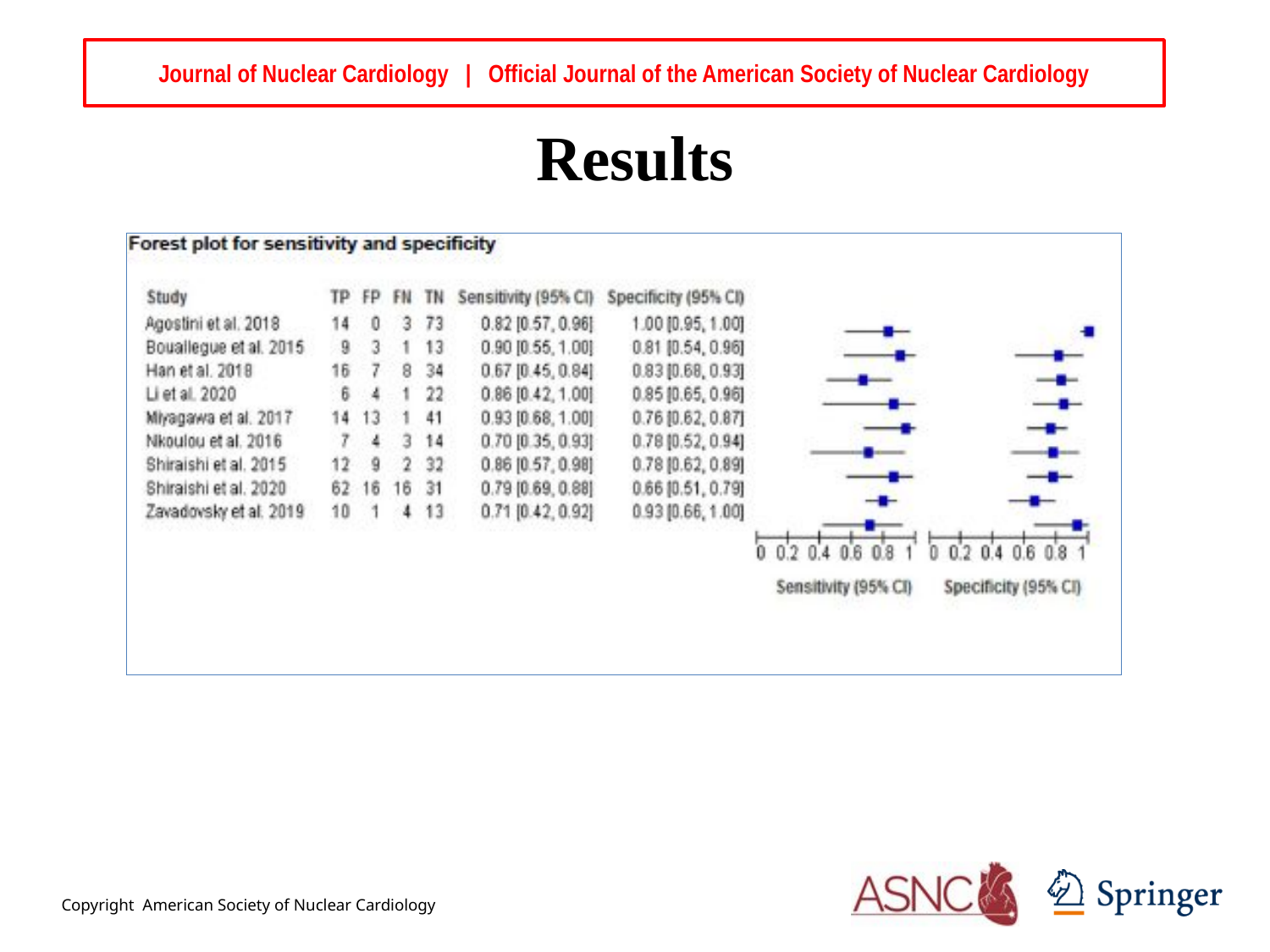

Journal of Nuclear Cardiology | Official Journal of the American Society of Nuclear Cardiology
# Results
Copyright American Society of Nuclear Cardiology

## Slide 5
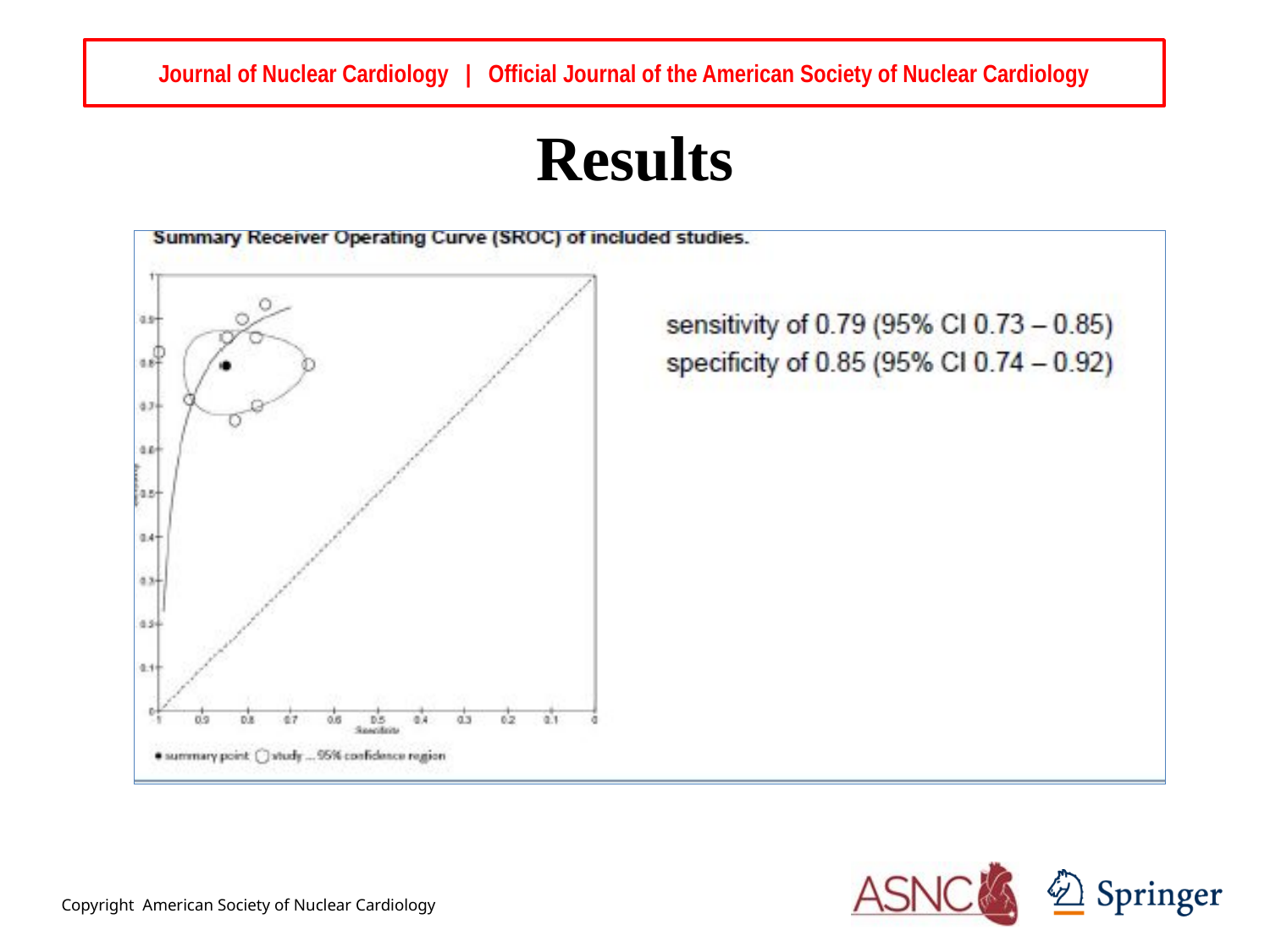

Journal of Nuclear Cardiology | Official Journal of the American Society of Nuclear Cardiology
# Results
Copyright American Society of Nuclear Cardiology

## Slide 6
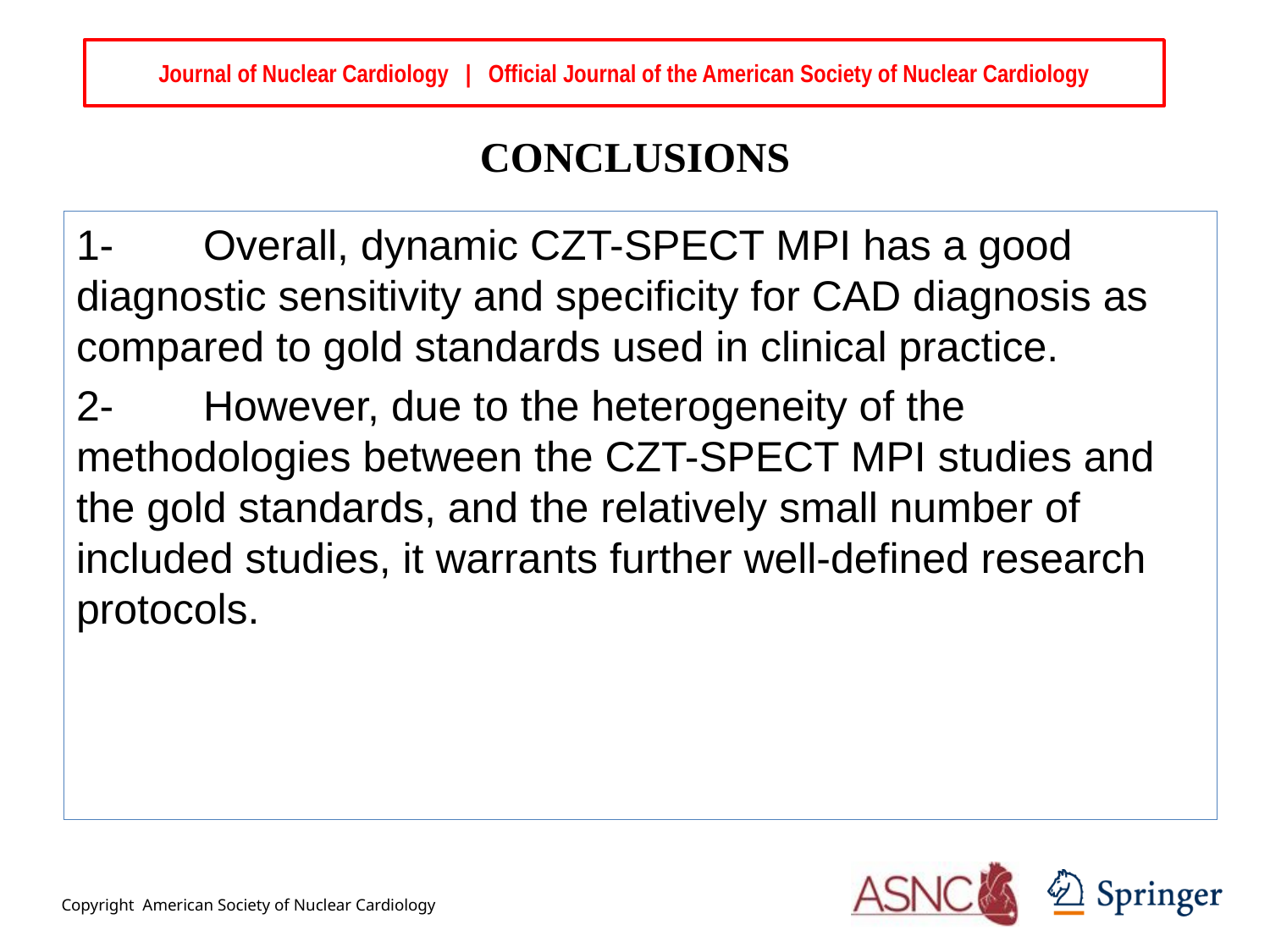

Journal of Nuclear Cardiology | Official Journal of the American Society of Nuclear Cardiology
# CONCLUSIONS
1-	Overall, dynamic CZT-SPECT MPI has a good diagnostic sensitivity and specificity for CAD diagnosis as compared to gold standards used in clinical practice.
2-	However, due to the heterogeneity of the methodologies between the CZT-SPECT MPI studies and the gold standards, and the relatively small number of included studies, it warrants further well-defined research protocols.
Copyright American Society of Nuclear Cardiology
